# Methylomic Changes of Autophagy-Related Genes by *Legionella* Effector Lpg2936 in Infected Macrophages

**DOI:** 10.3389/fcell.2019.00390

**Published:** 2020-01-28

**Authors:** Ahmed I. Abd El Maksoud, Dalia Elebeedy, Nasser H. Abass, Ahmed M. Awad, Ghada M. Nasr, Tamer Roshdy, Hany Khalil

**Affiliations:** ^1^Industrial Biotechnology Department, Genetic Engineering and Biotechnology Research Institute, University of Sadat City, Sadat City, Egypt; ^2^College of Biotechnology, Misr University for Science and Technology (MUST), 6th of October City, Egypt; ^3^Department of Molecular Biology, Genetic Engineering and Biotechnology Research Institute, University of Sadat City, Sadat City, Egypt; ^4^Molecular Diagnostics Department, Genetic Engineering and Biotechnology Research Institute, University of Sadat City, Sadat City, Egypt

**Keywords:** *Legionella pneumophila*, autophagy, Lpg2936 effector, N6-methyleadinine changes, macrophages

## Abstract

*Legionella pneumophila* (*L. pneumophila*) is a Gram-negative bacterium that infects the human respiratory tract causing Legionnaires’ disease, a severe form of pneumonia. Recently, rising evidence indicated the ability of *Legionella* to regulate host defense via its type 4 secretion system including hundreds of effectors that promote intracellular bacterial replication. The host defense against such invaders includes autophagic machinery that is responsible for degradation events of invading pathogens and recycling of cell components. The interplay between host autophagy and *Legionella* infection has been reported, indicating the role of bacterial effectors in the regulation of autophagy during intracellular replication. Here, we investigated the potential impact of *Legionella* effector Lpg2936 in the regulation of host autophagy and its role in bacterial replication using mice-derived macrophages and human lung epithelial cells (A549 cells). First, monitoring of autophagic flux following infection revealed a marked reduction of Atg7 and LC3B expression profile and low accumulation levels of autophagy-related LC3-I, LC3-II, and the Atg12–Atg5 protein complex. A novel methyladenine alteration was observed due to irreversible changes of GATC motif to G(6 mA) TC in the promoter region of Atg7 and LC3B indicated by cleaved genomic-DNA using the N6 methyladenine-sensitive restriction enzyme *Dpn*I. Interestingly, RNA interference (RNAi) of Lpg2936 in infected macrophages showed dramatic inhibition of bacterial replication by restoring the expression of autophagy-related proteins. This is accompanied by low production levels of bacterial-associated pro-inflammatory cytokines. Furthermore, a constructed Lpg2936 segment in the GFP expression vector was translocated in the host nucleus and successfully induced methyladenine changes in Atg7 and LC3B promoter region and subsequently regulated autophagy in A549 cells independent of infection. Finally, treatment with methylation inhibitors 5-AZA and (2)-Epigallocatechin-3-gallate (EGCG) was able to restore autophagy-related gene expression and to disrupt bacterial replication in infected macrophages. This cumulative evidence indicates the methylation effect of *Legionella* effector Lpg2936 on the host autophagy-related molecules Atg7 and LC3B and subsequent reduction in the expression levels of autophagy effectors during intracellular replication of *L. pneumophila.*

## Introduction

*Legionella pneumophila* is a Gram-negative bacterium that replicates in macrophages and causes a severe form of pneumonia called Legionnaires’ disease (LD) (Horwitz, 1983). Phagocytes degrade engulfed bacterial invaders via delivering them into the lysosomal compartment, however, a virulent strain of *Legionella* can avoid lysosomal fusion and ensures intracellular replication (Newton et al., 2010; Xu and Luo, 2013). *L. pneumophila* blazes a variety of intracellular communication by transferring more than 300 effectors through its secretion system, Icm/Dot type-IV, which modulates cellular immune response including cell signal transduction, autophagic machinery, apoptosis, and cytokine secretion (Hempstead and Isberg, 2013; Nevo et al., 2014). For instance, *Legionella* effector RomA is the first identified molecule with a methyltransferase domain that can epigenetically modify the host chromatin landscape through histone acetylation to modulate gene expression and to ensure bacterial replication (Rolando et al., 2013). Other *Legionella* effectors play a critical role in the regulation of signal transduction cascades and vesicular trafficking such as LetAS-RsmYZ-CsrA regulated effectors (Nevo et al., 2014). However, the function of *Legionella* effector Lpg2936 is not known, but a recent bioinformatics analysis indicated a complex between Lpg2936 and RNA methyltransferases (MTase) that is required to catalyze the transferring of a methyl group from S-adenosyl-L-methionine (SAM) to RNA molecules (Pinotsis and Waksman, 2017). Noteworthy, *L. pneumophila-*mediated inhibition of cellular autophagy, as a protective response, following infection has been observed through the regulatory function of *Legionella* effector RavZ on autophagy proteins conjugation system (Choy et al., 2012). Autophagy is a cellular process in which double-membraned vesicles are formed to surround the cytosolic defective contents by recruiting specific autophagy associated proteins (ATGs) and deliver these contents to the lysosomes for degradation (Klionsky et al., 2012). The elongation of autophagosomal vesicles requires the conjugation of ubiquitin-like proteins Atg12 and Atg8 (the microtubule-associated protein, LC3) to the substrates Atg5 and phosphatidylethanolamine (PE), respectively (Geng and Klionsky, 2008). Two isoforms of LC3 have been identified, the cytosolic isoform of LC3, unlipidated protein, which is referred to as LC3-I, and the membraned isoform of LC3, lipidated protein, which is known as LC3-II (Tanida et al., 2008). The unlipidated LC3-I is activated by the protease Atg4B through exposing a C-terminal glycine residue. Both conjugation reactions required E1-like activating enzyme, Atg7, while distinct E2-like enzymes, Atg3 and Atg10, are utilized for lipidated LC3-II and the Atg12–Atg5 complex, respectively (Fujita et al., 2008a). The Atg12–Atg5 conjugate associates with Atg16L to serve an E3-like enzyme for the transfer of LC3-I to PE (Fujita et al., 2008b). Rising evidence suggested that the lipidation of LC3 is required for elongation of the autophagosomal membrane, autophagosome formation, and lysosomal fusion (Tsuboyama et al., 2016). Importantly, autophagy is an immediate cellular response to *Legionella* infection and other pathogens, recognizing the bacterial inclusions as cargo for the lysosomal degradation system (Amer and Swanson, 2005; Abdelaziz et al., 2015). Activation of autophagy has been further connected with modulation of the cellular innate immune response, regulation of programmed cell death, and alteration of pro-inflammatory cytokine response (Levine et al., 2011; Deretic et al., 2013; Caution et al., 2015; Khalil et al., 2019). In atherosclerosis, the dysfunction of the autophagy process has been connected with the accumulation of pro-inflammatory cytokines and the development of cardiovascular disorder (Miyazaki and Miyazaki, 2017; Khalil et al., 2019). Recent evidence indicated that hypermethylation and disturbance of autophagy-related genes LC3B and Atg5 are associated with aging-related events (Rubinsztein et al., 2011; Khalil et al., 2016). Furthermore, activation of autophagy (xenophagy) is essential to engulf intracellular invaders forming autophagosomes and removing them through the lysosome degradation system (Escoll et al., 2016). Recently, the rescue of autophagy as a therapeutic strategy becomes highly considered in a variety of human diseases and medical disorders such as cardiovascular diseases, neurodegenerative diseases, and aging (Khalil et al., 2016, 2019; Fujikake et al., 2018). The emergence of autophagy as an anti-invader process encourages the examination of its role in the pathogenesis of *L. pneumophila* infection. Therefore, in the current study, we further investigated the role of *Legionella* effector Lpg2936 in the regulation of autophagic machinery as an important key for intracellular replication and the potential activation of the autophagy process during *Legionella* infection as a therapeutic strategy.

## Materials and Methods

### Mice-Derived Macrophages

Mice 4 weeks old (C57BL/6) were housed in the Ohio State University College of Medicine and were obtained from Jackson Laboratories. Bone marrow-derived macrophages were isolated and propagated as previously described (Zhang et al., 2008; Khalil et al., 2016). Accordingly, bone marrow cells were proliferated and differentiated in cell-culture dishes using Dulbecco’s Modified Eagles Medium (DMEM) in the presence of macrophage colony-stimulating factor (M-CSF) which is secreted by L929 cells and was used in the form of L929-conditioned medium.

### Cell Line

Human lung epithelial cells (A549 cells) (CCL-185, ATCC-LGC) were cultured in DMEM (Sigma-Aldrich, United Kingdom) (Invitrogen, Germany) supplemented with 2 mM L-glutamine, 2 mM sodium pyruvate, and 10% v/v bovine serum albumin (BSA). Cell culture was incubated at 37°C in a humidified 5% CO_2_ incubator and subcultured by using Trypsin/EDTA solution (Sigma-Aldrich) (Khalil et al., 2017b, 2018).

### Bacterial Growth and Infection

*Legionella pneumophila* JR32 strain was grown on buffered charcoal yeast extract (BCYE) and was incubated at 37°C for 3 days. The bacterial colonies were then individually resuspended in 5 ml of *Legionella* buffered yeast extract (BYE) medium supplemented with L-cysteine, ferric nitrate, and thymidine and allowed to continue to grow for another 2 days at 37°C. Growth concentration was carried out using a spectrophotometer, and the bacterial growth at an optical density (OD) of 600 nm was selected for infection of mice-derived macrophages at a multiplicity of infection (MOI) equal to 10. The inactive bacteria were prepared by heating the active *Legionella* at 95°C for 15 min (Amer et al., 2006; Abu Khweek et al., 2013).

### Cell Viability and Chemical Treatment

The MTT cell viability assay kit (Abcam, ab211091) was used to determine the live cell numbers and the inhibition percent of 5-AZA and EGCG on cell viability upon 24 h post-treatment. Therefore, mice-derived macrophages were seeded in 96-well plate at a density of 5 × 10^4^ cells per well and were incubated overnight at a CO_2_ incubator. Then, the cells were treated with the indicated concentrations of each inhibitor (0–300 μM) for 24 h. The serum-containing media in 96-well plates was replaced with serum-free media containing MTT reagent and the plates were incubated for 3 h at 37°C. Then, the MTT solvent was added to each well and incubated for 15 min. At last, the cells containing the formazan product were photometrically quantified at 590 nm. The inhibition rate (%) was calculated as (the mean absorbance values for control - the mean absorbance values for sample/the mean absorbance values for control) × 100 (Abd El Maksoud et al., 2019). For chemical treatment, macrophages derived from mice were seeded in six-well plates in a concentration of two million cells per well. One day later, cells were treated with 50 μM of EGCG or 5-AZA and were incubated for 2 h pre-infection. Other macrophages were treated with DMSO, which served as control. Then, the treated cells were infected with *Legionella* MOI of 10 and were incubated for different time points (2, 4, 8, and 24 h).

### Colony-Forming Units (CFU) Assay

Mice-derived macrophages were seeded in four different plates of 24-well plates in a concentration of 5 × 10^4^ cells per well. Chemical pre-treated macrophages were infected with *Legionella* MOI of 10. Then, cells and infectious media were collected at different time points (2, 24, 48, and 72 h) and diluted in PBS. One hundred microliters of diluted samples were plated in charcoal yeast extract plates (CYE). Three days later, the number of colonies was accounted and the growth curve of infected macrophages was performed using an Excel sheet.

### RNA Isolation and qRT-PCR

Total RNA from mice-derived macrophages and A549 cells was isolated by using TriZol (Invitrogen, United States), chloroform, and isopropanol and was purified using RNA purification kit (Invitrogen, United States). cDNA was prepared from total RNA using QuantiTech Reverse Transcriptase Kit (Qiagen, United States) according to the manufacturer’s protocol. Fold changes in steady-state mRNA of LC3B, Atg7, DNA methyltransferases (DNMTs), and pro-inflammatory cytokines were quantified using the QuantiTect SYBR Green PCR Kit (Qiagen, United States) and oligonucleotides specific for each gene (Table 1). Levels of GAPDH gene expression were used for normalization. The following PCR parameters were used to detect fold changes in gene expression: 95°C for 4 min, 40 cycles (95°C for 30 s, 62°C for 30 s, 72°C for 30 s), and the reaction was held at 4°C. The PCR data were analyzed as previously described using ΔΔCt equations (Tazi et al., 2016; Khalil et al., 2017b).

**TABLE 1:** Oligonucleotides sequences used for detection of steady state mRNA for the indicated genes.

Description	Primer sequences 5′-3′
LC3B-sense	AGAGTCGGATTCGCCGCCGCA
LC3B-antisense	GACGGCATGGTGCAGGGATCT
Atg7-sense	AGAGAGCTGTGGTTGCCGGAA
Atg7-antisense	GGGATCCTGGACTCTCTAAA
DNMT1-sense	CCCATGCATAGGTTCACTTCCTTC
DNMT1-antisense	TGGCTTCGTCGTAACTCTCTACCT
DNMT2-sense	CATACAATGCCCGTGTGAGTTCTTAAGG
DNMT2-antisense	CGTGTGTCTAAATGGCTTGAGTACAGT
DNMT3-sense	TGCAATGACCTCTCCATTGTCAAC
DNMT3-antsense	GGTAGAACTCAAAGAAGAGGCGG
IL1-α- sense	GAGATGCCTGAGACACCCA
IL1-α- antisense	GTGCACCAGTTTTCGTTCCT
IL1-β- sense	GCACGATGCACCTGTACGAT
IL1-β- antisense	CACCAAGCTTTTTTGCTGTGAGT
IL-6- sense	GCTCCTGGTGATGGCTACTG
IL-6- antisense	TGTTTGCAGAGGTGAGTGGT
TNF-α- sense	GAAGAGCTCCCAAATTGCCT
TNF-α- antisense	GCTACAACATGAGCTACTGGC
GAPDH-sense	TGGCATTGTGGAAGGGCTCA
GAPDH-antisense	TGGATGCAGGGATGATGTTCT

### Western Blot Investigation

Denatured proteins were electrophoresed using the vertical Bio-Rad Mini-Protean II electrophoresis system. One hundred nanograms of protein sample was loaded in 12% sodium dodecyl sulfate-polyacrylamide gel electrophoresis (PAGE). The electrophoresed proteins were then transferred onto nitrocellulose membranes (Millipore, MA, United States), followed by 2 h incubation at room temperature (RT) with rabbit polyclonal anti-*Legionella* (Invitrogen, United States), or rabbit monoclonal anti-LC3 (Sana Cruz, United States) or rabbit monoclonal anti-Atg5–Atg12 complex, which were individually diluted 1:1000 in the milk-blocking buffer. After washing, the membranes were incubated for another 2 h at RT with diluted secondary antibody donkey anti-rabbit that conjugated with horseradish peroxidase. The enhanced chemiluminescence system (ECL, Amersham) was used to detect the protein band signals, after soaking the membrane for almost 1 min with the Western blotting detection reagents (the chemiluminescence solutions 1 and 2) that were mixed 1:1 (Amersham, Piscataway, NJ, United States). Housekeeping protein β-actin protein was used as a loading control (Khalil et al., 2016, 2017b). The quantification of Western blot patterns was done using ImageJ software, which indicated the relative band intensity that was normalized to the intensity of housekeeping protein band.

### Fluorescence Confocal Microscopy Assay

Mice-derived macrophages and A549 cells were plated with a density of 5 × 10^4^ cells/well in 24-well plates that supplemented with poly-L-lysine coverslips and were incubated overnight. Following transfection and/or Legionella infection, the coverslips were blocked and fixed by using 500 μl of cold methanol for 3 min at RT. The fixed cells were then permeabilized using 5% goat serum and 0.1% Triton X-100 for 30 min. The cells were then incubated overnight at 4°C with rabbit polyclonal antibody against Legionella protein (Invitrogen, United States) (1:100 dilution). After washing the cells were incubated in the dark for 1 h at RT with goat anti-rabbit conjugated Alexa Fluor 488 secondary antibody (Invitrogen, United States) (1:1000 dilution). For LC3 staining, the cells were incubated in the dark for 1 h at RT with the previously indicated antibody against LC3 (1:500 dilution) followed by 1 h incubation with Alexa Fluor 594 goat anti-rabbit (Invitrogen, United States) (1:1000 dilution). Finally, the cells were stained for 15 min at RT with 1 μg/ml of DAPI, a DNA fluorescent dye. Coverslips were then placed onto glass slides using mowiol and samples were examined by a Leica TCS-SP laser scanning confocal microscopy. Photomicrographs were processed using Adobe Photoshop 7.0M (Adobe Systems) and Microsoft PowerPoint (Khalil et al., 2016; Tazi et al., 2016). The quantification of confocal images was performed using ImageJ software in which the percentage of the cells with more than five puncta of each protein was quantified within a field of approximately 500 cells.

### DNA Isolation and Epigenetic Analysis

The genomic DNA was isolated from infected macrophages and transfected A549 cells by using a DNA purification kit (Qiagen, United States) according to the manufacturer’s protocol. Genomic DNA was digested with the methyladenine-sensitive restriction enzyme *Dpn*I (Thermo Scientific, United States), which preferentially cleaves methylated adenine at the position GATC by using 5 units of the enzyme to 1 μg of DNA and incubation for 2 h at 37°C (Yao et al., 2017). The cleaved products were then electrophoresed on 1% agarose gel using 1 × TBE buffer, and DNA fragments were visualized with a long-wave (320 nm) UV *trans*-illuminator (Luo et al., 2016). The cleaved products were also used to amplify the 3′-UTR of both Atg7 and LC3B using specific oligonucleotides with reverse complementary sequences of GATC motif in the reverse primers (Table 1). The qRT-PCR was used to quantify the relative amplified autophagy-related molecules by using the following PCR parameters: the initial denaturation step (95°C for 5 min) and then 40 cycles (94°C for 20 s, 58°C for 30 s and 72°C for 15 s). The *Dpn*I-cleaved DNA of LpDME-transfected A549 cells were eluted from agarose gel using a GeneJET gel extraction kit (Thermo Scientific, United States). Sequencing analysis of eluted fragments was performed using the Illumina NovaSeq 6000 sequencing system. The DNA alignment between sequence data and the 3′-UTR of Atg7 and LC3B was done by using an online tool (Corpet, 1988).

### Cloning Strategy

To construct LpDME overexpression vector, GFP plasmid with CMV promoter was used to clone *Legionella* Lpg2936 cDNA downstream of GFP fragment. To generate the construct pGFP-LpDME, Lpg2936 cDNA was amplified using the following specific oligonucleotides containing restriction sites specific for Sac1 and Pst1: Sac1-For-5**′**-gagctcatgttatagatcacctccggcag-3**′** and Pst1-Rev-5**′**-ctgcagttggctgtgagaacaatacgtatct-3**′**. To isolate Lpg2936 coding sequence, total RNA was isolated and purified from infected macrophages using an RNA purification kit (Invitrogen, United States). cDNA was synthesized from total RNA using Lpg2936-Pst1-Reverse primer and the following reagents: 2 μl of 10 × RT buffer (Promega), 2 μl of RNA (500 ng/μl), 4 μl of dNTPs (25 mM), 1 μl of RNase inhibitor (10 U/μl) (Promega), and 1 μl of M-MuLV reverse transcriptase (200 U/μl) (Promega) up to 20 μl final volume using RNase-free water. Then, the reagents were incubated for 2 h at 42°C followed by 10 min at 90°C to inactivate the enzyme. The synthesized cDNA was then used to amplify Lpg2936 fragment using the above specific primers and conventional PCR. PCR product was then digested with Sac1 and Pst1 as well as pEGFP plasmid as recommended under NEB conditions. The open pEGFP vector was eluted from the agarose gel and was incubated with digested fragments with 5 U of T4 DNA ligation and 4 μl of 5 × ligation buffer (Promega), 2 μl from DNA (100 mM) and 2 μl from the vector (1 μM) in the final volume of 20 μl. The products were transformed into competent *Escherichia coli* strain by heat shock (42°C for 45 s). Maxi-Prep kit (Qiagen, United States) was used to isolate and purify the constructs, which were then tested for the right sequences and the right orientation using the restriction sites map in the right construct prepared by cloning manager program.

### Transfection Protocol

A549 cells were maintained in the complete DMEM medium and were overnight cultured in either 24-well plates with coverslips or 6-well plates with a confluency of 70–80%. A549 cells were then transfected with 1 μg/ml GFP-LpDME construct using Lipofectamine LTX (Invitrogen, United States), according to the manufacturer’s instructions. The transfected cells were incubated for 24 h followed by qRT-PCR, immunofluorescent, and immunoblotting assays. Mice-derived macrophages seeded in six-well plates with confluency of 60–70% were transfected with the respective siRNA against *Legionella* effector Lpg2936 (5**′**′-UUGCGTCAAUAACGGCGCCAAA-3**′**) or anti-Luciferase (5**′**-AACUUACGCUGAGUACUUCGA-3**′**), which served as control (Khalil et al., 2016). According to the manufacturer’s instructions, the cells were transfected with 500 ng of siRNA/well using 20-μl HyperFect transfection kits (Qiagen, United States) that were prepared in 500 μl of optimum media. Transfected cells were incubated for 48 h and then were infected with *L. pneumophila* (MOI of 10) for 3 days. The knockdown efficiency of Lpg2936 and the relative expression of autophagy-related Atg7 and LC3B genes in addition to bacterial infectivity (CFU) were monitored daily in transfected and infected cells. The immunoblotting and immunofluorescent assays for the Atg5–Atg12 protein complex and accumulated LC3B were assessed at day 2 post-infection.

### Enzyme-Linked Immunosorbent Assay (ELISA)

The interleukin 1 (IL-1α and β) assay kits (Mouse ab199076 and 100704, respectively) were used to quantify the concentration of both interleukins in the fluid media of RNAi-transfected and -infected macrophages. For quantitative measurement of IL-6 and TNF-α, the mouse ELISA kits (Invitrogen, United States) were used. According to the manufacturer’s protocol, the standard curve was prepared and color-dependent ILs concentration was measured at 450 nm. Finally, the concentrations of targeted interleukins were calculated according to the equation: final concentration = (amount/sample volume) × dilution factor (Khalil et al., 2017a, 2018).

### Statistical Analysis

Q-RT-PCR data were assessed using the previously described ΔΔCt equations. Accordingly, fold changes in steady-state mRNA of targeted genes are equal to 2^–ΔΔct^ (Khalil et al., 2017b). Student two-tailed *t*-test was used to determine the differences in analyzed data. ^∗^*P* < 0.05 was considered statistically significant and ^∗∗^*P* < 0.01 was considered highly significant.

## Results

### *Legionella* Infection Is Accompanied by a Reduction of Expression of Autophagy Effectors

**FIGURE 1:**
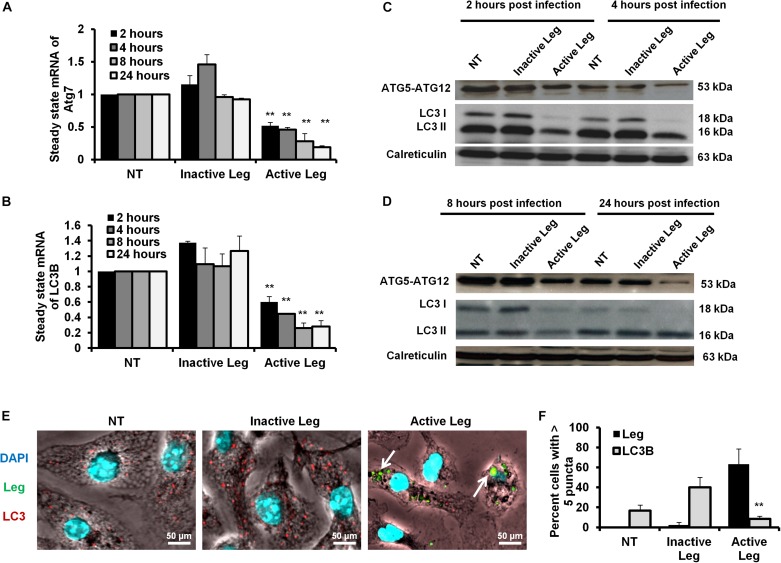
Depletion of autophagosome formation in response to *Legionella* infection. **(A)** The fold change in steady-state mRNA of autophagy Atg7 during the indicated time points upon *Legionella* infection of mice-derived macrophages compared with control-infected cells. **(B)** Fold change in steady-state mRNA of LC3B in *Legionella-*infected macrophages related to control-infected cells during the indicated time points. GAPDH-mRNA was used as an internal control for the qRT-PCR test. **(C,D)** Immunoblotting assays indicate Atg5–Atg12 protein complex, LC3-I, and LC3-II in infected macrophages for different time points compared with control-infected cells. Calreticulin served as an internal control. **(E)** Immunofluorescent assay represents accumulated LC3B protein (red) and *Legionella* protein biomarker (green) in mice-derived macrophages in response to *Legionella* infection. 4′,6-Diamidino-2-phenylindole, dihydrochloride (DAPI) was used for chromosome counterstaining (blue). **(F)** Scoring of the percentage of macrophages harboring more than five puncta in infected cells compared with other control cells using ImageJ software. Data are representative of 500 cells. Error panels shown represent three independent experiments displaying similar results. Comparison of groups for statistical differences was done using two-tailed Student *t* test. ^∗^*P* ≤ 0.05 and ^∗^^∗^*P* ≤ 0.01.

Cellular autophagy plays a dual role during infection. It is either anti-microbial or an initiator for pro-replication. To investigate the impact of autophagy during *Legionella* infection, we infected mice-derived macrophages with *L. pneumophila* JR32 with a MOI of 10. The effect of *Legionella* infection on autophagy progress was monitored at different time points following infection (2, 4, 8, and 24 h). Steady-state mRNA of autophagy-related genes Atg7 and LC3B were detected by qRT-PCR while the expression profile of its corresponding proteins was investigated using immunoblotting and immunofluorescent assays. Interestingly, in comparison with inactive *Legionella*, *Legionella* infection showed a significant reduction of both Atg7 and LC3B mRNA in a time-dependent manner following infection (Table 2 and Figures 1A,B). Similarly, in Western blot, infection with active *Legionella* reduced the expression profile of the Atg5–Atg12 protein complex as well as lipidated LC3-II and unlipidated LC3-I during bacterial infection (Figures 1C,D). Quantification of protein band intensity validated the significant reduction of the autophagy-related Atg5–Atg12 complex, LC3-I, and LC3-II following infection (Supplementary Figures S1A–D). These findings were further confirmed by immunofluorescent assay, which revealed a marked inhibition of LC3 protein puncta in infected cells (Figure 1E). Quantification analysis of immunofluorescent images revealed significant reduction of LC3 puncta in infected macrophages when compared with cells infected with inactive *Legionella* (Figure 1F). These data firstly indicate the low expression level of autophagy-unconjugated LC3 following *Legionella* infection and suggest the effect of *Legionella* invasion on autophagy-related gene expression.

**TABLE 2:** Statistical analysis of autophagy related genes expression in infected macrophages compared with non-infected cells.

Genes	Conditions	Fold changes	Standard deviation	Student two tails *t*-test	*P*-values
Atg7	2 h. p. i.	0.51	0.05	0.005	≤0.01**
	4 h. p. i.	0.46	0.03	0.001	≤0.01**
	8 h. p. i.	0.27	0.12	0.014	≤0.01**
	24 h. p. i.	0.19	0.02	0.003	≤0.01**
LC3B	2 h. p. i.	0.6	0.07	0.015	≤0.01**
	4 h. p. i.	0.44	0.009	0.001	≤0.01**
	8 h. p. i.	0.25	0.07	0.004	≤0.01**
	24 h. p. i.	0.28	0.07	0.005	≤0.01**

**Highly significant.

### Rescue of Autophagic Machinery via RNAi of *Legionella* Effector Lpg2936

**FIGURE 2:**
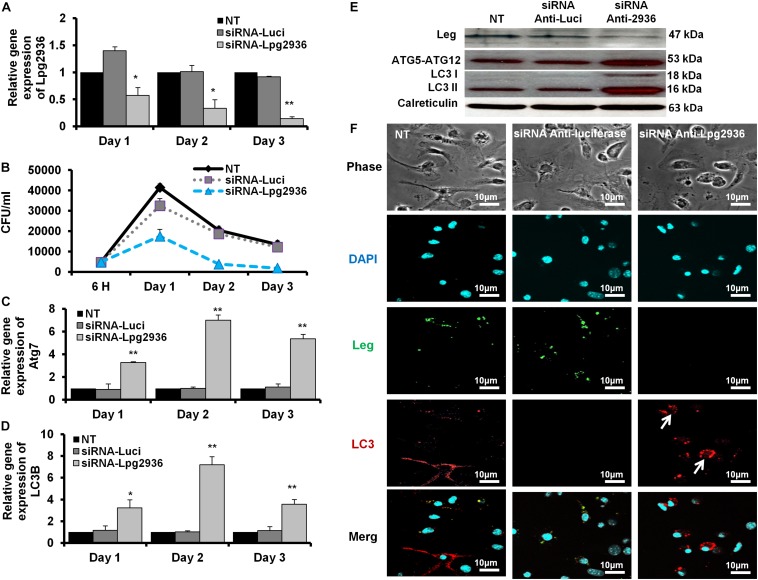
Autophagosome formation and bacterial replication in Lpg2936-knockdown cells. **(A)** Quantification of Lpg2936 steady-state mRNA in mice-derived macrophages that represented the knockdown efficiency during indicated time points following infection in comparison with control transfection (siRNA-Luci) using qRT-PCR test. **(B)** Colony-forming unit (CFU) assay of *Legionella* replication in siRNA-Lpg2936 transfected macrophages during the indicated time points post-infection compared to control-transfected and non-transfected cells. **(C,D)** Relative expression of autophagy-related genes Atg7 and LC3B in siRNA-transfected and -infected macrophages during the indicated time points using qRT-PCR. **(E)** Immunoblotting assays represent *Legionella* indicator protein, Atg5–Atg12 protein complex, LC3-I, and LC3-II in siRNA-transfected and -infected macrophages. Calreticulin served as an internal control. **(F)** Immunofluorescent assay represents accumulated LC3B puncta (red) and *Legionella* protein biomarker (green) in murine-derived macrophages in response to siRNA transfection-induced Lpg2936 knockdown. DAPI was used for chromosome counterstaining (blue). Error bars reveal the SD of three independent experiments. Student two-tailed *t* test was used for statistical differences of Ct values in different groups. ^∗^*P* ≤ 0.05 and ^∗^^∗^*P* ≤ 0.01.

*Legionella* effector, Lpg2936, is a ribosomal RNA protein that has been recently reported as a potential methylation effector via its complex in a crystal structure with S-adenosyl-L-methionine (Pinotsis and Waksman, 2017). Therefore, we investigated the potential restoration of autophagy-related genes in infected macrophages via RNAi of Lpg2936. Accordingly, mice-derived macrophages were transfected with either siRNA that targets Lpg2936 (anti-Lpg2936) or siRNA against Luciferase (anti-Luci), which served as control. Two days later, transfected macrophages were infected with *Legionella* MOI of 10 followed by detection of autophagosome formation and bacterial replication. The knockdown efficiency of Lpg2936 was monitored by qRT-PCR at different time points (24, 48, and 72 h). The result showed that mRNA of Lpg2936 was gradually reduced in a time-dependent manner and the knockdown efficiency was almost 90% after 72 h of transfection (Figure 2A). Further, during the earlier stage of infection in control-transfected macrophages, bacterial replication increased, reaching a peak within 24 h, and then decreased, while bacterial replication in macrophages transfected with anti-Lpg2936 siRNA was significantly lower throughout the infection period indicated by colony-forming units per milliliter (CFU/ml) (Figure 2B). Steady-state mRNA of both Atg7 and LC3B were significantly increased (up to sixfold increase) in macrophages transfected with siRNA-Lpg2936 during the infection period in comparison with control-transfected macrophages, which revealed a sustained level of these mRNAs at the indicated time points (Table 3 and Figures 2C,D). To determine whether targeting of *Legionella* effector Lpg2936 can disturb bacterial replication and can restore autophagic machinery in infected macrophages, we investigated the expression profile of *Legionella* indicator protein and autophagy-related proteins using immunoblotting analysis and immunofluorescent assay. Noteworthy, the expression of *Legionella* indicator protein was markedly decreased in Lpg2936-knockdown macrophages compared with control-transfected cells. Interestingly, both conjugated and unconjugated LC3 protein and the Atg5–Atg12 protein complex were markedly increased in transfected macrophages with siRNA against Lpg2936 when compared to control-transfected macrophages (Figure 2E). Quantification analysis of protein band intensity showed a significant reduction of *Legionella* indicator protein accompanied by increasing levels of autophagy-related proteins in Lpg2936-knockdown cells (Supplementary Figure S2A). To evaluate whether restoring the expression of autophagy-related proteins increases the basal autophagic activity in Lpg2936-knockdown macrophages, the number of macrophages exhibiting more than five puncta of accumulated LC3 was quantified using confocal microscopy. Importantly, the higher accumulated LC3 puncta were quantified in transfected macrophages with siRNA against Lpg2936 when compared with control-transfected macrophages (Figure 2F and Supplementary Figure S2B). Further, the quantification analysis of *Legionella* protein revealed decreasing levels in Lpg2936-knockdown macrophages (Supplementary Figure S2B). These findings indicate that the expression profile of autophagy family member proteins is reduced in the presence of *Legionella* infection and can be restored in *Legionella*-infected macrophages when *Legionella* effector Lpg2936 is depleted.

**TABLE 3:** Statistical analysis of indicated genes expression in infected microphages that were pre-transfected with siRNA against Lpg2939 compared with control infected cells.

Genes	Conditions	Fold changes	Standard deviation	Student two tails *t*-test	*P*-values
Lpg2936	Day 1	0.57	0.14	0.05	≤ 0.05*
	Day 2	0.33	0.15	0.02	≤ 0.05*
	Day3	0.14	0.03	0.008	≤ 0.01**
Atg7	Day 1	3.2	0.08	0.006	≤ 0.01**
	Day 2	6.9	0.47	0.003	≤ 0.01**
	Day3	5.3	0.38	0.003	≤ 0.01**
LC3B	Day 1	3.2	0.74	0.05	≤ 0.05*
	Day 2	7.1	0.75	0.007	≤ 0.01**
	Day3	3.5	0.42	0.01	≤ 0.01**

*Significant and **Highly significant.

### Downregulation of Legionella Lpg2936 Significantly Reduces Pro-inflammatory Cytokine Secretion

**FIGURE 3:**
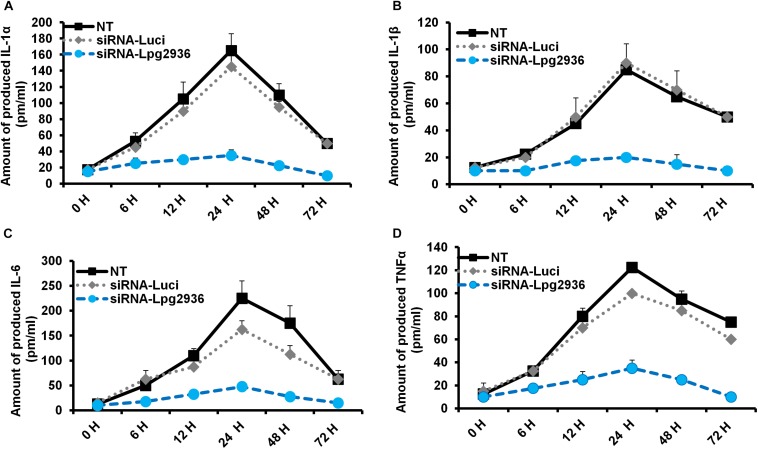
Levels of produced pro-inflammatory cytokines in Lpg2936-knockdown and infected cells. The concentration of pro-inflammatory cytokines (pm/ml) that are produced in the fluid media of infected macrophages in response to siRNA transfection against *Legionella* effector Lpg2936 compared with control transfection (anti-Luciferase): **(A)** The concentration of IL-1α. **(B)** The concentration of IL-1β. **(C)** The concentration of IL-6. **(D)** The concentration of TNF-α.

*Legionella* infection of macrophages induces a variety of pro-inflammatory cytokines during bacterial replication (McHugh et al., 2000; Jung et al., 2016). To determine whether RNAi of *Legionella* effector Lpg2936 affects cytokine secretion, the concentration of IL-1α, IL-1β, IL-6, and TNF-α was monitored in culture media of infected macrophages using ELISA. Both control-transfected and -infected macrophages showed rising levels of IL-1α, IL-1β, IL-6, and TNF-α at 24 h compared with other time points. In contrast, macrophages transfected with siRNA against Lpg2936 showed very low or undetectable levels of indicated cytokines during infection (Figure 3). As indicated in Figure 3A, IL-1α protein was markedly elevated (up to 160 pm/ml) in culture medium collected from control-transfected and -infected macrophages, but not in culture media collected from macrophages that were pre-transfected with anti-Lpg2936 siRNA. During *Legionella* infection, the production level of IL-1β was significantly reduced in the supernatant of transfected macrophages with siRNA against Lpg2936 compared to control-transfected macrophages (Figure 3B). Furthermore, following *Legionella* infection, IL-6 and TNF-α were evidently reduced in the supernatant of transfected macrophages with anti-Lpg2936 siRNA, while control-transfected macrophages showed a marked increase of both cytokines in a time-dependent manner (Figures 3C,D). Together, these data reveal the biological impact of *Legionella* effector Lpg2936 in the regulation of pro-inflammatory cytokine secretion during bacterial replication. Further, regulation of cytokine secretion via targeting autophagy-reducer Lpg2936 could provide evidence for the regulatory effect of autophagy in the accumulation and production of cytokines from infected macrophages.

### Exogenous Expression of GFP-Tagged Lpg2936 Reduces Autophagy Independently of Infection

**FIGURE 4:**
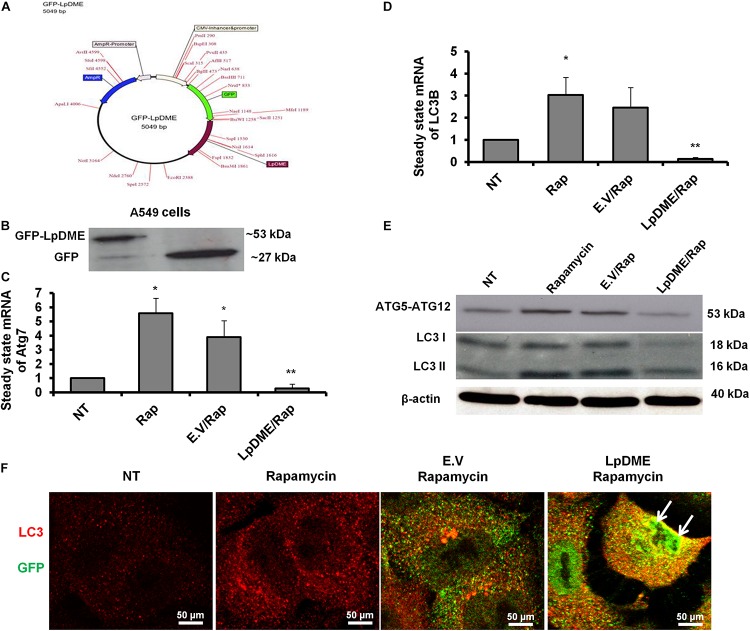
Lpg2936-independent regulation of autophagy. **(A)** Schematic representation of GFP-LpDME constructs revealing cloned full-length cDNA of Lpg2936 downstream GFP cassette. **(B)** Immunoblotting analysis of resulting GFP and Lpg2936 protein levels in transfected A549 cells using specific antibodies against GFP. **(C,D)** Relative gene expression of Atg7 and LC3B, respectively, in transfected and rapamycin-treated cells compared with control-treated cells using qRT-PCR assay. **(E)** Immunoblot analysis detecting Atg5–Atg12 protein complex and conjugated LC3 protein in transfected and rapamycin-treated cells compared with control-treated cells. **(F)** Fluorescent confocal images represent the considerable levels and localization of GFP-Lpg2936 fusion protein (green) and accumulated LC3 puncta (red) in transfected cells upon rapamycin treatment. Error panels showed the SD of three independent experiments. Student two-tailed *t* test was used for statistical analysis of Ct values in different groups. ^∗^*P* ≤ 0.05 and ^∗^^∗^*P* ≤ 0.01.

To address whether *Legionella* effector Lpg2936 can regulate autophagy progress independently of infection, we constructed GFP fusion protein of Lpg2936 into novel expression vector called *L. pneumophila* DNA Methylation Effector (LpDME) (Figure 4A). The tagged protein expression, interaction, and localization were investigated by immunofluorescent microscopy and Western blot in transfected A549 cells. A549 cells were transfected with either developed LpDME construct or GFP-empty vector (E.V) which served as control. Then, transfected cells were treated with rapamycin as an autophagy-inducer agent and were incubated for 24 h. By using specific antibody against GFP, GFP-Lpg2936 was detected by immunoblotting assay, which revealed the original GFP fragment at almost 27 kDa in control-transfected cells and the tagged protein band at almost 53 kDa in LpDME-transfected cells (Figure 4B). Further, gene expression of both Atg7 and LC3B indicated by quantification of steady-state mRNA was significantly downregulated in LpDME-transfected cells following rapamycin treatment compared to control-transfected cells (Table 4; Figures 4C,D). Meanwhile, the relative expression of autophagy-related LC3A showed comparable levels in transfected cells indicating the selective regulation of both Atg7 and LC3B (Supplementary Figure S3A). Similarly, at the protein level, the expression of autophagy effectors, Atg5–Atg12 protein complex, and both conjugated and unconjugated LC3 was significantly reduced in LpDME-transfected cells even after rapamycin treatment compared with other control cells (Figure 4E and Supplementary Figure S3B). Lastly, the cellular localization of Lpg2936 protein was analyzed using immunofluorescent assay. Interestingly, GFP fluorescent signal was detected in the nucleus in most transfected cells with LpDME, indicating that GFP-Lpg2936 tagged protein can reside into the nucleus as expected. By contrast, the expression of non-tagged GFP in control-transfected cells was only detected in the cytoplasm, indicating that the presence of GFP tagged to the Lpg2936 protein did not alter its ability to reside in the nucleus (Figure 4F). Together, these data indicate that Lpg2936 resides into the nucleus and can regulate the autophagic process induced by rapamycin treatment independent of infection.

**TABLE 4:** Statistical analysis of autophagy related genes expression in rapamycin (Rap) treated A549 cells that were pre-transfected with LpDME construct in comparison with control transfected cells.

Genes	Conditions	Fold changes	Standard deviation	Student two tails *t*-test	*P*-values
Atg7	Rap	5.58	1.06	0.03	≤0.05*
	E.V/Rap	3.9	1.15	0.05	≤0.05*
	LpDME/Rap	0.1	0.06	0.002	≤0.01**
LC3B	Rap	3.3	0.8	0.05	≤0.05*
	E.V/Rap	2.4	0.9	0.15	>0.05
	LpDME/Rap	0.15	0.05	0.002	≤0.01**

*Significant and **Highly significant.

### *Legionella* Infection Induces Accumulation of Adenine N6 Methylation in the Promoter Region of Atg7 and LC3B

**FIGURE 5:**
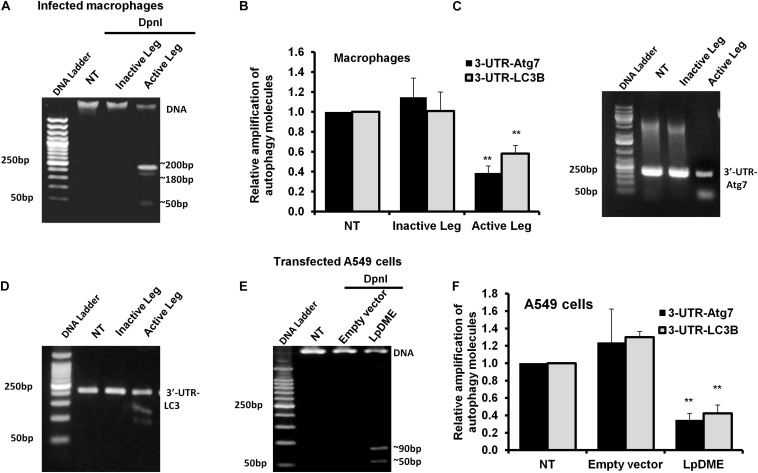
Adenine N6 methylation changes in 3**′**-UTR of Atg7 and LC3 induced by *Legionella* effector Lpg2936. **(A)** Agarose gel electrophoresis represents genomic DNA that was isolated from *Legionella*-infected macrophages and digested with methylation-dependent *Dpn*I for 2 h. **(B)** Fold change of amplified autophagy-related molecules, 3**′**-UTR-Atg7 and 3**′**-UTR-LC3B, in *Dpn*I-cleaved DNA product for infected cells using qRT-PCR. **(C,D)** Agarose gel electrophoresis represents the amplified autophagy-related molecules 3**′**-UTR-Atg7 and 3**′**-UTR-LC3B, respectively. **(E)** Agarose gel electrophoresis reveals *Dpn*I-cleaved genomic DNA isolated from LpDME-transfected A549 cells in comparison with cleaved genomic DNA isolated from control-transfected cells. **(F)** Fold change of amplified autophagy-related molecules, 3**′**-UTR-Atg7 and 3**′**-UTR-LC3B, in *Dpn*I-cleaved DNA product for transfected cells using qRT-PCR. Error panels indicate the SD of three independent experiments. Student two-tailed *t* test was used for statistical differences of Ct values in different groups. ^∗^*P* ≤ 0.05 and ^∗^^∗^*P* ≤ 0.01.

Methylation on the sixth position of adenine base (6 mA) is a novel chemical modification on DNA molecules that has been recently reported (Yao et al., 2017; Xiao et al., 2018). Interestingly, the sequence analysis of the promoter region for both autophagy Atg7 and LC3B exhibits a canonical repeated sequence of GATC motif that can be tested with the N6-methyladenine-senstive restriction enzyme, *Dpn*I. Accordingly, to interrogate the methyladenine changes in DNA of infected macrophages, genomic DNA was isolated from infected cells and was incubated with the *Dpn*I enzyme for 2 h. Interestingly, the agarose gel electrophoresis of cleaved DNA from *Legionella*-infected cells showed three digested fragments with a molecular size of approximately 200, 180 and 50 bp when compared to cleaved DNA from other control cells (Figure 5A). The *Dpn*I-cleaved DNA was then used in qRT-PCR to amplify the 3**′**-UTR fragment of Atg7 and LC3B using specific oligonucleotides with a reverse complementary sequence of GATC motif in the reverse primers. Noteworthy, the relative amplification of the 3**′**-UTR of both Atg7 and LC3B was significantly decreased in macrophages infected with active *Legionella* in comparison with the cells infected with inactive *Legionella* and non-infected cells (NT) (Figure 5B). This finding was further confirmed by agarose gel electrophoresis of qRT-PCR products which revealed low-intensity band of the 3**′**-UTR-Atg7 and 3**′**-UTR-LC3B (Figures 5C,D), respectively. To evaluate if *Legionella* effector Lp2936 induces accumulation of methyladenine changes in the 3**′**-UTR region of Atg7 and LC3B, genomic DNA was isolated from A549 cells transfected with either LpDME construct or empty vector. The purified DNA from transfected cells was then incubated with *Dpn*I for 2 h followed by agarose gel electrophoresis. Interestingly, two digested fragments with a molecular size of approximately 90 and 50 bp were detected in cells transfected with LpDME construct (Figure 5E). The sequence analysis of these fragments showed a similarity region of about 60 nucleotides and 80 nucleotides with the 3**′**-UTR of Atg7 and LC3B, respectively (Supplementary Figure S4). Further, in the qRT-PCR assay of cleaved DNA, the relative amplification of the 3**′**-UTR of both Atg7 and LC3B was significantly decreased in LpDME-transfected A549 cells when compared with the empty vector-transfected cells (Figure 5F). These findings strongly suggest the possible methyladenine changes in autophagy-related Atg7 and LC3B in response to *Legionella* infection by its Lp2936 effector in mice-derived macrophages and A549 cells. To exclude the role of DNMTs in such methyladenine changes, the relative expression of different isotypes of DNMTs was determined in infected macrophages by using qRT-PCR. The steady-state mRNA of DNMT isoforms showed negligible differences in *Legionella-*infected macrophages in comparison with control-infected cells (Supplementary Figure S5). These data suggest the absence of the direct effect of DNMTs during *Legionella* infection.

### Restoration of Autophagy by Methylation Inhibitors of Infected Macrophages

**FIGURE 6:**
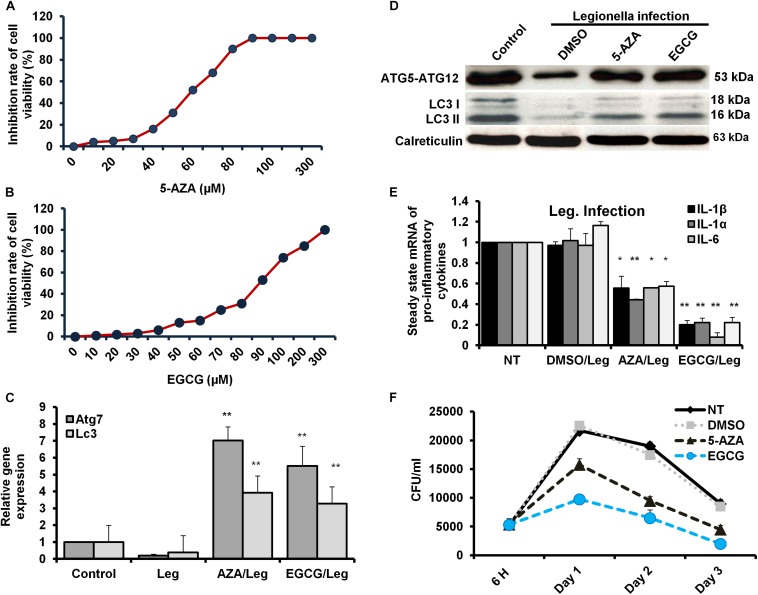
Resting of *Legionella* replication via restoring autophagy-related genes in macrophages in response to methylation inhibitor treatment. **(A,B)** Cell viability rate of mice-derived macrophages that were overnight-treated with the indicated concentration of either 5-AZA or EGCG by using MTT kit. **(C)** Relative gene expression of autophagy-related Atg7 and LC3 in *Legionella*-infected macrophages that were pre-treated with 50 μM of either 5-AZA or EGCG compared with control-infected cells using qRT-PCR. **(D)** Immunoblotting assays represent autophagy-related Atg5–Atg12 protein complex, LC3-I, and LC3-II in infected macrophages that were pre-treated with 50 μM of either 5-AZA or EGCG compared with DMSO-treated and infected cells. Calreticulin served as an internal control. **(E)** Fold change in steady-state mRNA of the indicated pro-inflammatory cytokines in infected macrophages that were pre-treated with 50 μM of either 5-AZA or EGCG compared with DMSO-treated and infected cells using qRT-PCR. **(F)** CFU of *Legionella* particles in mice-derived macrophages that were pre-treated with 50 μM of either 5-AZA or EGCG during the indicated time points compared with DMSO-treated and infected cells. Error panels indicate the SD of three independent experiments. Student two-tailed *t* test was used for statistical differences of Ct values in different groups. ^∗^*P* ≤ 0.05 and ^∗^^∗^*P* ≤ 0.01.

The *Legionella* effector Lpg2936 is required for epigenetic modification of autophagy-related molecules via methylation of (6 mA). Therefore, we tested the ability of methylation inhibitors such as 5-azacytosine (5-AZA) and (2)-Epigallocatechin-3-gallate (EGCG) to restore the expression of autophagy effectors. First, to assess the cytotoxic effect of the indicated methylation inhibitors, the inhibition rate of cell viability was monitored in treated macrophages with different concentration of 5-AZA or EGCG using an MTT kit. As shown in Figures 6A,B, treatment with 80 μM of 5-AZA showed almost 50% inhibition of cell viability, while EGCG showed 50% inhibition of cell viability at a concentration of 90 μM. Therefore, the mice-derived macrophages were treated with 50 μM of either 5-AZA or EGCG for 2 h before *Legionella* infection. Then, the bacterial replication and the basal autophagy activity have been assessed in 24 h following infection. We observed that the relative expression of both Atg7 and LC3 at RNA levels was strongly increased in infected cells that were pre-treated with either 5-AZA or EGCG compared with control-infected cells (Table 5 and Figure 6C). Treatment with methylation inhibitors exhibit marked autophagy increased expression of the Atg5–Atg12 protein complex and both conjugated and unconjugated LC3 proteins (Figure 6D and Supplementary Figure S6). Furthermore, the methylation inhibitors reduced the expression levels of steady-state mRNA of associated pro-inflammatory cytokines including IL-1β, IL-1α, IL-6, and TNF-α (Table 5 and Figure 6E). Interestingly, pre-treatment of infected cells with the inhibitors successfully inhibits bacterial replication during the time course experiment compared with control-infected cells as indicated by the number of CFU/ml (Figure 6F). These results indicate that the methylation inhibitors 5-AZA and EGCG can block *Legionella* infection via restoring autophagy-related genes expression in infected macrophages.

**TABLE 5:** Statistical analysis of indicated genes expression in infected macrophages that were pre-treated with methylation inhibitors compared with DMSO treated and infected cells.

Genes	Conditions	Fold changes	Standard deviation	Student two tails *t*-test	*P*-values
Atg7	Control/Leg	0.2	0.09	0.006	≤ 0.01**
	5-AZA/Leg	7.02	0.81	0.009	≤ 0.01**
	EGCG/Leg	5.52	1.17	0.003	≤ 0.01**
LC3B	Control/Leg	0.45	0.1	0.015	≤ 0.01**
	5-AZA/Leg	4.04	0.16	0.001	≤ 0.01**
	EGCG/Leg	3.15	0.18	0.003	≤ 0.01**
IL1-β,	Control/Leg	0.96	0.03	0.3	>0.05
	5-AZA/Leg	0.55	0.11	0.03	≤ 0.05*
	EGCG/Leg	0.2	0.04	0.001	≤ 0.01**
IL1-α,	Control/Leg	1.01	0.17	0.86	>0.05
	5-AZA/Leg	0.44	0.006	0.008	≤ 0.01**
	EGCG/Leg	0.22	0.043	0.01	≤ 0.01**
IL-6	Control/Leg	0.96	0.03	0.3	>0.05
	5-AZA/Leg	0.55	0.04	0.005	≤ 0.01**
	EGCG/Leg	0.07	0.05	0.001	≤ 0.01**
TNF-α,	Control/Leg	1.16	0.09	0.12	>0.05
	5-AZA/Leg	0.57	0.01	0.006	≤ 0.01**
	EGCG/Leg	0.22	0.04	0.008	≤ 0.01**

*Significant and **Highly significant.

## Discussion

**FIGURE 7:**
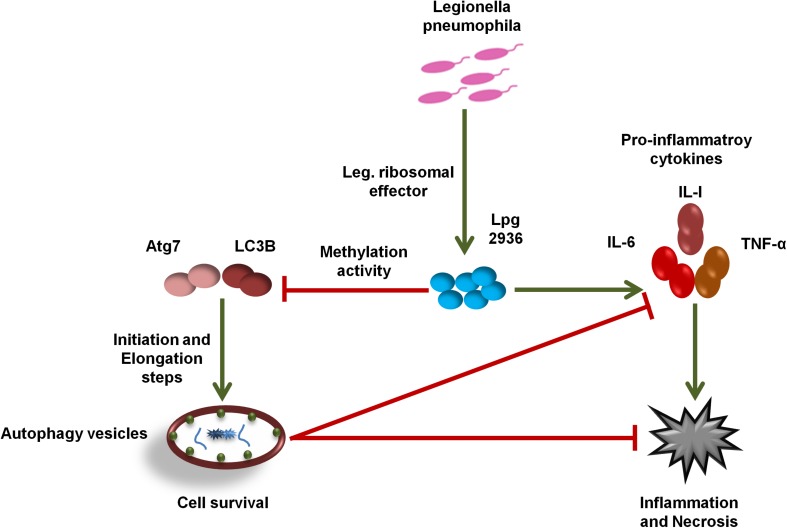
Schematic model represents the function of Lpg2936 enzyme during *Legionella* replication. To modulate host defense before infection, *L. pneumophila* submits a specific agent known as Lpg2936 effector, which translocates in the host nucleus and begins its methylation activity. The first methylated molecules are autophagy-related; Atg7 and LC3B resulted in marked inhibition of autophagosome formation and high production levels of *Legionella*-associated pro-inflammatory cytokines. Such events modulate the cellular immune response and promote intracellular replication of *L. pneumophila.*

In the current work, we demonstrate the direct role of the *L. pneumophila* effector Lpg2936 in the methyladenine changes of autophagy-related Atg7 enzyme and LC3B-asscoited protein to ensure intracellular bacterial replication. In particular, we present that the *Legionella*-secreted effector Lpg2936 targets the promoter regions and epigenetically modifies GATC motif to G(6 mA)TC in autophagy-related molecules Atg7 and LC3B. Therefore, the relative expression of autophagy-related genes, Atg7 and LC3B, were gradually decreased in a time-dependent manner following infection. Likewise, the levels of unlipidated and lipidated LC3 in addition to the autophagy-related Atg5–Atg12 protein complex were reduced during infection as presented by immunoblotting and immunofluorescent assays. To determine whether *Legionella* effector Lpg2936 is involved in the regulation of host autophagy, infected mice-derived macrophages were pre-transfected with siRNA that specifically targets Lpg2936. Notably, targeting of Lpg2936 by RNAi restored the expression of autophagy-related genes Atg7 and LC3B during infection and increased the protein level of the Atg5–Atg12 complex, unconjugated LC3-I, and conjugated LC3II proteins when compared to control-transfected and -infected macrophages. Because of the potential methylation activity of Lpg2936 protein in autophagic molecules, we investigated its role independent of infection. Our results showed that the expression of both Atg7 and LC3B at the RNA level was significantly decreased in A549 cells transfected with LpDME construct even in the presence of rapamycin treatment, which is a potent stimulator of autophagy. This result demonstrates that the low expression of Atg7 enzyme and LC3B protein exerted by *Legionella* effector cannot be remedied by rapamycin treatment. Importantly, detection of GFP-tagged *Legionella* effector signals in the nucleus by immunofluorescent microscopy reveals the ability of the constructed protein to translocate into the nucleus of transfected A549 cells, which showed low accumulation puncta of autophagy-related LC3 protein. To address if the currently available methylation inhibitors can avoid the activity of *Legionella* effector Lpg2936, mice-derived macrophages were treated with either 5-AZA or EGCG before *Legionella* infection. Upon infection, the expression of autophagy-related genes Atg7 and LC3B at the RNA level was significantly restored in treated cells when compared with untreated cells. Likely, protein levels of LC3-I, LC3-II, and the Atg5–Atg12 complex were markedly increased in both 5-AZA- and EGCG-treated cells. In addition, treatment with methylation inhibitor showed competitive inhibition on bacterial replication and its associated pro-inflammatory cytokine secretion. Noteworthy, the *Legionella* flagellin effectors and peptidoglycan-associated lipoprotein (PAL) are recognized by host cells toll-like receptor 2 (TLR2), TLR5, and TLR9, which activate the downstream nuclear factor NF-κB, inflammasome, and caspase-1 to induce pro-inflammatory cytokine production and restrict bacterial infection (Liu and Shin, 2019). Controlling the production of pro-inflammatory cytokines has been reported through the activation of inflammasomes inducing lysosome–autophagosome fusion (Abdelaziz et al., 2015). This clearly shows the role of autophagy and inflammasomes in confronting *Legionella* infection and regulating the release of pro-inflammatory cytokines from infected cells. Therefore, *L. pneumophila* works to block the autophagic response to increase the multiplication of bacteria and exhausts the neighboring cells by increasing pro-inflammatory cytokine secretion. Lpg2936 is one of the *Legionella* effectors that transmitted to host cells through *Legionella* secretion system Dot/Icm type IV. Recently, the potential methylation activity of the *Legionella* effector Lpg2936 has been reported based on the analysis of its crystal structure conducted with S-adenosyl L-methionine as a cofactor (Pinotsis and Waksman, 2017). Based on our findings, *Legionella* effector Lpg2936 seems to interact as a transcription factor that translocates in the host nucleus and recognizes GATC motifs in the promoter regions of autophagy-related Atg7 enzyme and LC3B. By S-adenosyl L-methionine-cofactor, Lpg2936 can induce the methyladenine changes of the Atg7 enzyme which is required for the conversion of unlipidated LC3-I to lipidated LC3-II, the membraned isoform of autophagosomes vesicles. Moreover, Lpg2936 induces methyladenine changes in the LC3B promoter region affecting the production of unlipidated LC3-I and subsequently the formation of lipidated isoform of LC3 and the autophagy-related protein complex Atg5–Atg12. Unlike Lpg2936 effector, the *Legionella* enzyme RomA was demonstrated as a methyltransferase enzyme that is able to epigenetically modify host chromatin and suppress host gene expression to ensure bacterial replication (Rolando et al., 2013). Further, the role of RavZ effector in the regulation of autophagic flux via inhibition of LC3 conjugation system has been reported during *Legionella* replication (Choy et al., 2012). Overall, *Legionella* has many effectors with multiple activities that can regulate host gene expression and its related cellular signaling to promote intracellular replication of *Legionella*. Noteworthy, recent cumulative evidence indicates that processing of host autophagy, as host defense, regulates enteric bacterial infection and avoids inflammatory events associated with host cell death (Chang et al., 2013; Jo et al., 2013). Another evidence suggests that the residence within autophagosomes supply the nutritional needs for *Legionella* replication as long as *Legionella* can avoid lysosomal fusion (Joshi and Swanson, 2011). Alternatively, intracellular inclusions of *Legionella* have been observed to prevent the activities of autophagy conjugation enzyme, Atg7, and recruitment of LC3 protein during autophagosome formation, however, the exact mechanism was not provided (Amer and Swanson, 2005). Because of the important role played by host autophagy in erasing invaders, each pathogen tries to overcome or exploit this phenomenon. For instance, *Helicobacter pylori* VacA protein can disturb autophagosome formation via attacking the endoplasmic reticulum and induces programmed cell death in infected cells (Zhu et al., 2017). Meanwhile, exploitation of xenophagy vesicles to protect bacterial inclusion from lysosomal degradation has been observed during intracellular replication of *Chlamydia trachomatis* (Al-Younes et al., 2004). Herein, we provide evidence suggesting that the *Legionella*-secreted effector Lpg2936 has a major role in the regulation of autophagosome formation through epigenetic alteration of the promoter region for autophagic enzymes Atg7 and LC3 (Figure 7). Meanwhile, we further confirmed the ability of methylation inhibitors, such as 5-AZA and EGCG agents, to avoid methylation activity of *Legionella* effectors, indicating that these agents can be considered as potential candidates for therapeutical strategy of Legionaries disease.

## Data Availability Statement

All datasets generated for this study are included in the article/Supplementary Material.

## Ethics Statement

All mice were housed in the Ohio State University, College of Medicine and mice-derived macrophages were performed at Amer laboratory with approval and in accordance with regulations and guidelines from the Institutional Animal Care and Use Committee (IACUC) at the Ohio State University (Columbus, OH, United States).

## Author Contributions

HK and AIA established the study design and provided the research strategy. HK, AIA, DE, NA, AMA, TR, and GN assessed the experiments and provided the data analysis. HK prepared and wrote the manuscript. All authors read and approved the manuscript.

## Conflict of Interest

The authors declare that the research was conducted in the absence of any commercial or financial relationships that could be construed as a potential conflict of interest.
