# Supplementary material for: Methylomic Changes of Autophagy-Related Genes by Legionella Effector Lpg2936 in Infected Macrophages
Source: Front Cell Dev Biol. 2020 Jan 28;7:390. doi: 10.3389/fcell.2019.00390 (PMC6999459; doi:10.3389/fcell.2019.00390)
Supplement: Supplementary file 1 [file Data_Sheet_1.pdf]

Supplementary Material

Supplementary figure 1: Quantification analysis of autophagy-related proteins expression in infected macrophages at different time points.

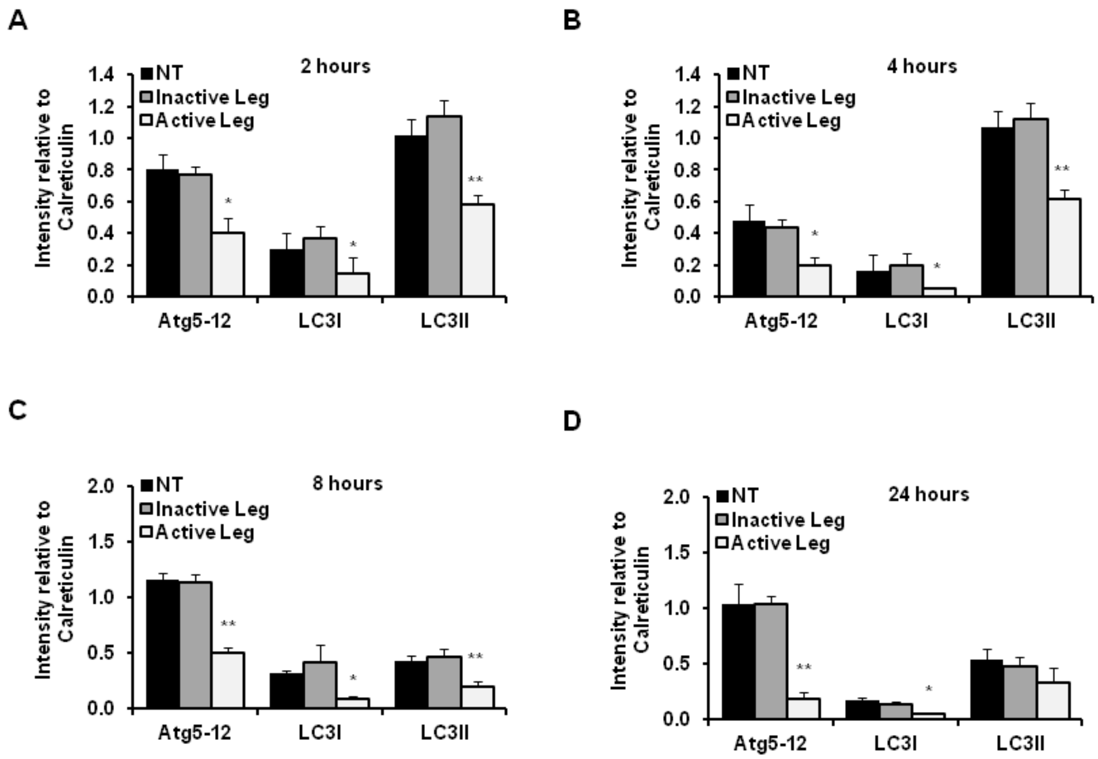

Densitometry analyses of western blot bands of Atg5-12, LC3I, and LC3II that were normalized to their respective housekeeping protein bands (calreticulin) at the indicated time points using ImageJ software. Error bars showed represent 3 independent experiments displaying similar results. Asterisks (*) indicates $P \leq 0.05$ and (**) $P \leq 0.01$

Supplementary figure 2: Quantification analysis of Legionella and autophagy-related proteins expression in transfected macrophages.

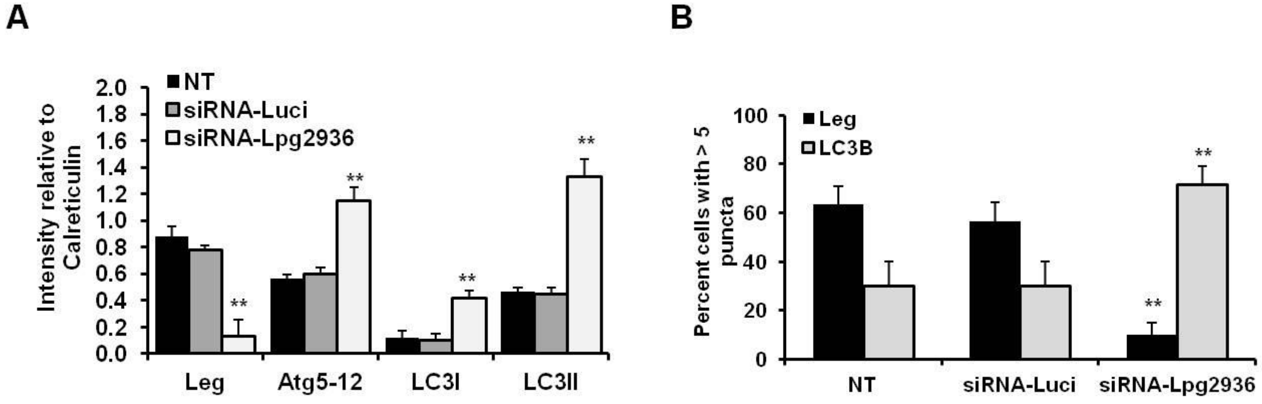

- **(A)** Densitometry analyses of western blot bands of Legionella protein, Atg5-12, LC3I, and LC3II that were normalized to calreticulin using ImageJ software. **(B)** Scoring of the percentage of macrophages harboring more than 5 puncta in transfected cells with siRNA against Luciferase or Lpg2936 in comparison with non-transfected (NT) macrophages using ImageJ software. Data are representative of 500 cells. Error panels indicate the SD bars of 3 independent experiments. Asterisks (*) indicates P ≤ 0.05 and (**) P ≤ 0.01

Supplementary figure 3: Quantification analysis of autophagy-related genes expression in transfected and rapamycin-treated A549 cells

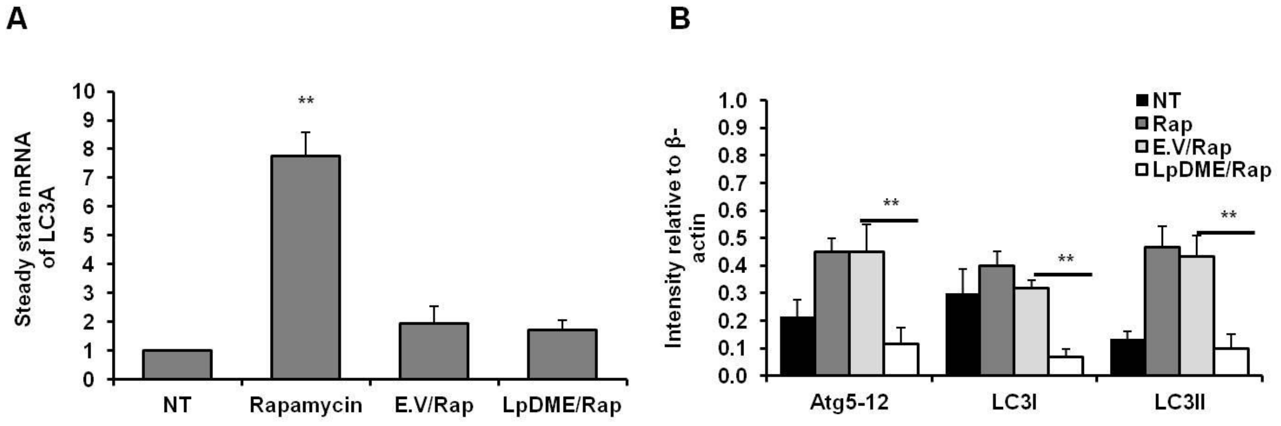

- **(A)** Fold change in steady-state mRNA of autophagy LC3A in rapamycin-treated A549 cells that were pre-transfected with either LpDME construct or GFP-empty vector. GAPDH-mRNA was used as an internal control for the qRT-PCR assay **(B)** Quantification analyses of western blot bands of Atg5-12, LC3I, and LC3II that were normalized to their respective housekeeping protein bands (β-actin) using ImageJ software. Error bars indicate the SD of 3 independent experiments. Asterisks (*) indicates P ≤ 0.05 and (**) P ≤ 0.01

Supplementary figure 4: Alignment between sequences analysis of DpnI-digested fragments and 3-UTR region of autophagy-related Atg7 and LC3B

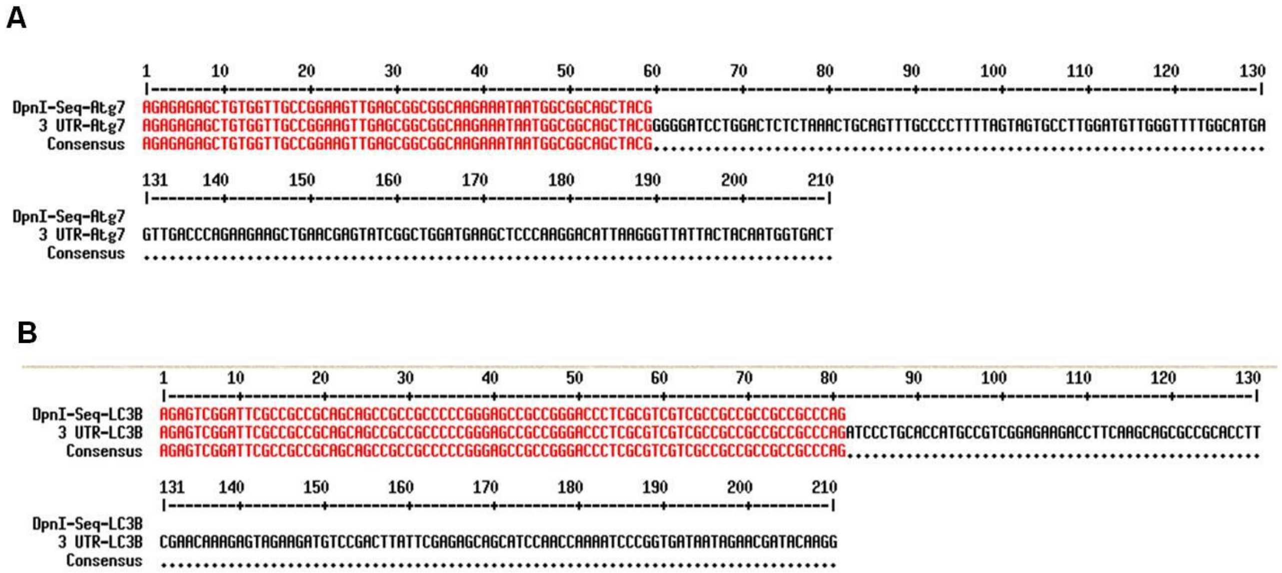

Identified region of similarity between DNA digested fragment of transfected A549 cells and 3'-UTR sequences of Atg7 (A) and 3'-UTR sequences of LC3B (B) by using the online tool; http://multalin.toulouse.inra.fr/multalin/multalin.html

Supplementary figure 5: Relative gene expression of DNMTs in infected macrophages

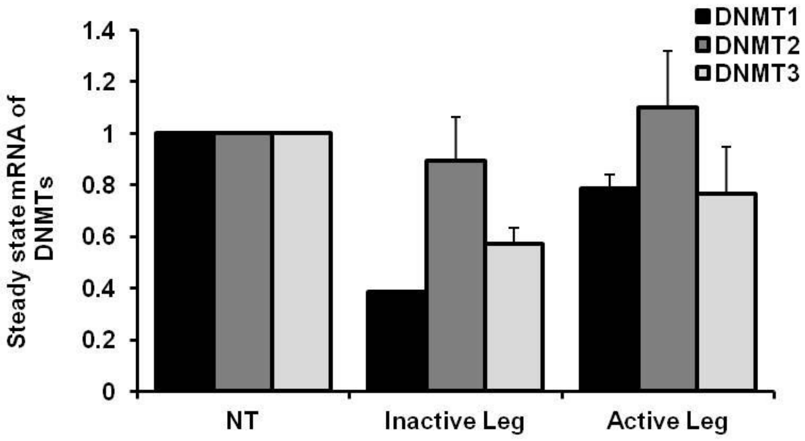

- Fold change in steady-state mRNA of the indicated DNMTs in infected macrophages compared with non-infected cells (NT) using qRT-PCR assay. GAPDH-mRNA was used as an internal control. Error bars showed the SD of 3 independent experiments.

Supplementary figure 6: Quantification analysis of autophagy-related proteins expression in infected macrophages that were pre-treated with the methylation inhibitors (5-AZA and EGCG).

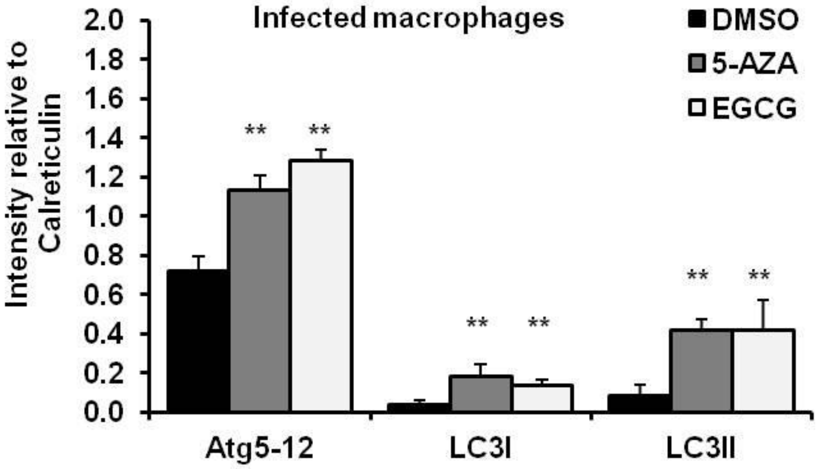

Densitometry analyses of western blot bands of Atg5-12, LC3I, and LC3II that were normalized to calreticulin using ImageJ software. Error bars indicate the SD of 3 independent experiments. Asterisks (*) indicates $P \leq 0.05$ and (**) $P \leq 0.01$
